# Meta-analysis and metaregression of risk factors associated with mortality in hip fracture patients during the COVID-19 pandemic

**DOI:** 10.1038/s41598-021-89617-2

**Published:** 2021-05-12

**Authors:** Firas J. Raheman, Djamila M. Rojoa, Jvalant Nayan Parekh, Reshid Berber, Robert Ashford

**Affiliations:** 1 The Leicester Royal Infirmary, University Hospitals of Leicester, Leicester, UK; 2 Nottingham University Hospitals, NHS Trust, Nottingham, UK; 3 Department of Trauma and Orthopaedics, Leicester Royal Infirmary, Balmoral Building, Infirmary Square, Leicester, LE1 5WW UK

**Keywords:** Diseases, Health care, Medical research, Risk factors

## Abstract

Incidence of hip fractures has remained unchanged during the pandemic with overlapping vulnerabilities observed in patients with hip fractures and those infected with COVID-19. We aimed to investigate the independent impact of COVID-19 infection on the mortality of these patients. Healthcare databases were systematically searched over 2-weeks from 1st–14th November 2020 to identify eligible studies assessing the impact of COVID-19 on hip fracture patients. Meta-analysis of proportion was performed to obtain pooled values of prevalence, incidence and case fatality rate of hip fracture patients with COVID-19 infection. 30-day mortality, excess mortality and all-cause mortality were analysed using a mixed-effects model. 22 studies reporting 4015 patients were identified out of which 2651 (66%) were assessed during the pandemic. An excess mortality of 10% was seen for hip fractures treated during the pandemic (OR 2.00, p = 0.007), in comparison to the pre-pandemic controls (5%). Estimated mortality of COVID-19 positive hip fracture patients was four-fold (RR 4.59, p < 0.0001) and 30-day mortality was 38.0% (HR 4.73, p < 0.0001). The case fatality rate for COVID-19 positive patients was 34.74%. Between-study heterogeneity for the pooled analysis was minimal (I^2^ = 0.00) whereas, random effects metaregression identified subgroup heterogeneity for male gender (p < 0.001), diabetes (p = 0.002), dementia (p = 0.001) and extracapsular fractures (p = 0.01) increased risk of mortality in COVID-19 positive patients.

## Introduction

The 2019 novel coronavirus (COVID-19) was declared as a global pandemic by the World Health Organisation (WHO) on the 11th March 2020^1^. The COVID-19 pandemic has led to a global surge in critically ill patients forcing hospitals to reallocate resources and potentially compromising the accessibility of essential care. Hip fractures constitutes a large proportion of emergency orthopaedic workload globally with approximately 1.66 million cases per year^2^. They represent the commonest injury sustained by patients over 50 years of age with an incidence of 1.1% in the USA and 1.6% in Europe^3^. As a result of COVID-19 there has been a reduction in the footfall of major orthopaedic trauma and activity related trauma^4^, however the incidence of fragility fractures has remained unchanged^5^. The pandemic has transformed the provision of orthopaedic services with multiple centres reducing their elective workload by up to 40% to restrict the spread of COVID-19 and safeguard healthcare resources^6^.

The advanced age, high frailty index and multiple comorbidities of hip fracture patients predispose them to peri-operative complications^7^ with a high 30-day mortality of 7–10% and a 1-year mortality of 37.1% for men and 26.4% for women^8,9,10^. Access to timely and high-quality care is necessary to achieve the best outcomes for patients. For those who survive, there is often a deterioration in both quality of life and independence level^7^. Many of the known risk factors for hip fracture overlap significantly with those associated with poor outcomes for COVID-19. These include male gender, diabetes mellitus, hypertension, chronic lung disease and old age^11^. Additionally, up to a third of patients have delayed seeking essential medical care due to the fear of contracting COVID-19 which may worsen outcomes in hip fracture patients as this is a well-established predictor of mortality^12,13^. Thus, provisions are required to ensure appropriate and timely management of these patients^14^.

Whilst attempts have been made to evaluate the mortality of hip fracture patients during COVID-19, these studies are limited by their search strategy and oversight in addressing sources of heterogeneity that may directly or indirectly impact the outcomes of hip fracture patients^15,16^. The aim of our systematic review, meta-analysis and meta-regression is to quantitatively assess the independent impact of COVID-19 on the mortality of hip fracture patients and identify characteristics predictive of poor outcomes. Further analysis of additional variables which may worsen prognosis of these patients is considered in view of a second and possible third ‘wave’ of COVID-19 until a definitive treatment of COVID-19 is available^17^.

## Methodology

The aim and methodology of this review was registered at the International Prospective Register of Systematic Reviews, PROSPERO, (CRD42020219709) and is reported in accordance with the Preferred Reporting Items for Systematic Reviews and Meta-analysis (PRISMA) and Meta-Analysis of Observational Studies in Epidemiology (MOOSE) standards^18,19^.

### Eligibility

All types of studies evaluating the direct and indirect impact of COVID-19 on mortality of hip fracture patients were considered. Letters, case reports, case series of less than 5 patients, review articles, and other grey literature such as conference abstracts and commentaries were excluded. Eligible participants were patients who were admitted having sustained a low-energy hip fracture either during the COVID-19 period or a comparative time-matched pre-COVID-19 period. Eligible studies reporting patient demographics, comorbidities, injury patterns with their respective management were included. Moreover, all eligible studies reported hospital quality measures such as length of stay (LOS), post-operative complications, inpatient and 30-day mortality and case fatality rate of COVID-19 infection.

### Information sources and search strategy

The literature search strategy was developed in collaboration with a senior information specialist and was performed over a 2-week period, 1st–14th November 2020. The Healthcare Databases Advanced Search (HDAS) interface developed by the National Institute for Heath and Care Excellence (NICE) was used to conduct a comprehensive search of the EMBASE, MEDLINE and EMCARE databases as well as the Cochrane Register of Studies (CRS) (CENTRAL) databases. A combination of controlled vocabulary and free text terms was used without any language constraints. The search strategy is presented in Supplementary Appendix 1.

### Selection process and data collection

Titles and abstracts were initially screened by two independent authors (FJR and DMR) and full-text articles based on eligibility and inclusion criteria were reviewed. Data was extracted by two review authors (FJR and DMR) using a spreadsheet. Author name, year of publication, type and design of study, study period, sample size, patient characteristics, COVID-19 status, comorbidities using the Charlson Comorbidity Index (CCI), social status, cognitive status and frailty scores using the Nottingham Hip Fracture Score (NHFS) or the Clinical Frailty Score (CFS). Outcomes in terms of mortality, post-operative complications and length of stay were also recorded.

The included studies in this review were performed over different time periods during this pandemic. Due to varying infection rates worldwide, we evaluated its impact as a possible contributor to inter-study heterogeneity. Through published data on the prevalence of COVID-19 infection and associated hospital occupancy^20,21^, we obtained estimates of the average 14-day COVID-19 positive cases (COVID-19 prevalence) and number of patients in-hospital with COVID-19 for each country included in the study period.

### Data items

The following data was collected from the included studies:

- Article (Author, year, journal of publication)
- Study design (Sample size, type of study)
- Study population and demographics (Age, gender, comorbidities)
- COVID-19 prevalence
- Trauma (Patterns, fracture type, management, type of fixation)
- Hospital quality measures (Length of stay, time to surgery, rehabilitation)
- Outcomes

### Outcomes and prioritization

Our outcome measures included all-cause mortality due to concurrence COVID-19 infection, excess mortality when comparing outcomes during pandemic and pre-pandemic controls, in-patient mortality and 30-day mortality.

### Risk of bias in individual studies

The critical appraisal for methodological quality was performed by two review authors independently (FJR and DMR) and discrepancies were resolved by a third author (RB). The Newcastle–Ottawa scale (NOS) for non-randomized studies was used, with a range of 0–9^22,23^.

### Synthesis and statistical analysis

A descriptive synthesis summarised study characteristic, patient demographics and reported outcomes. Where substantial heterogeneity in study design and population demographics occurred, a narrative review was used to analyse this data. Meta-analysis using a mixed effects model was only performed when no evidence of substantial design and study characteristic heterogeneity was found. We calculated excess mortality during the COVID-19 pandemic to evaluate its true impact on hip fracture mortality irrespective of the direct deaths caused by COVID-19 infection. Our aim was to capture COVID-19 deaths that were not correctly diagnosed or missed, in addition to indirect deaths from other causes attributable to the overall crisis. Moreover, mortality in all hip fractures testing positive for COVID-19 was compared to non-positive COVID-19 mortality and defined as all-cause mortality. Pooled dichotomous outcomes were analysed to obtain estimates of odds ratio (OR) or risk ratio (RR) and associated 95% confidence intervals (CI)^24^.

For late outcomes (e.g., 30-day mortality) a time-to-event data meta-analysis was performed using the inverse variance method to obtain summary hazard ratios (HR) with 95% CI. Based on a paper by Tierney et al.^25^ a mixture of direct (e.g. results from COX regression models or reported HR’s and 95% CI) or indirect methods (e.g. reported log-rank test p-value with events to patient ratios or estimates from published survival curves) was applied to calculate the individual study HR and standard error (SE) for outcome measures. A random-effects meta-regression was performed to assess potential sources of heterogeneity for studies reporting COVID-19 hip fracture case fatality and mortality rates^26^. Estimates for declared COVID-19 prevalence, hospital occupancy by country and known risk factors were incorporated into the meta-regression model.

### Sensitivity analyses

Sensitivity analysis was performed to evaluate the robustness of the observed outcomes and compare studies rated as low or moderate risk of bias and assess against potential confounders in all studies reporting adjusted and unadjusted results. A Newcastle–Ottawa Score (NOS) of 5 or more has been shown to be moderate or good quality rating of papers, hence this cut-off was used for sensitivity analysis^27^.

### Heterogeneity of included studies

Inter-study heterogeneity was assessed using calculated *X*^2^ and I^2^ statistic, whereby a *X*^2^ p-value < 0.05 and I^2^ < 50% suggested low heterogeneity. Publication bias was assessed using funnel plots for outcomes reported by 22 studies and Egger’s test assessed for small study effects^22,23^. All analyses were performed on STATA 16 (Stata-corp, College station, Texas, USA).

## Results

### Systematic review search results

**Figure 1:**
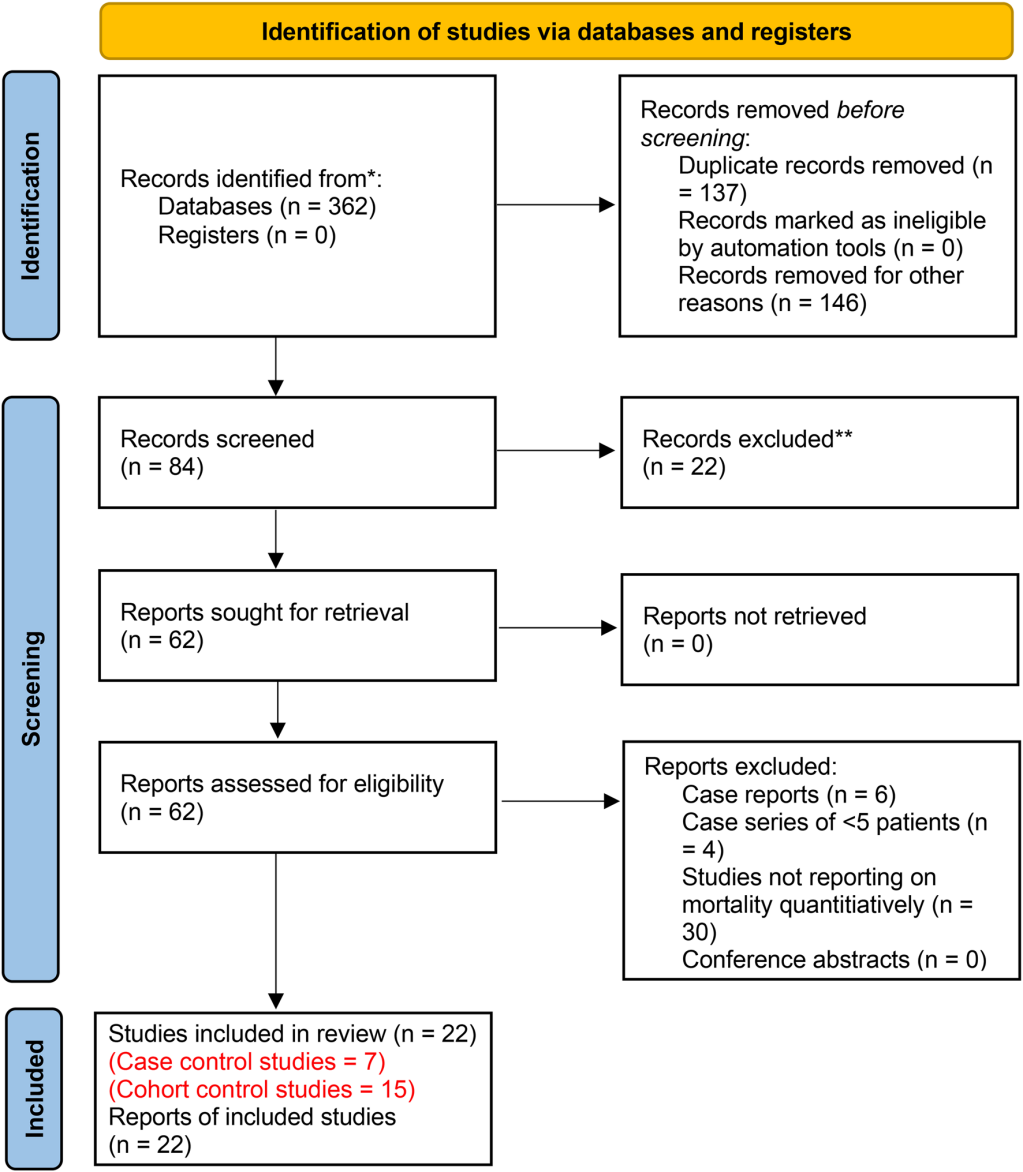
Shows the PRISMA diagram for the search strategy and study selection.

Through the search, 146 studies were obtained from Embase, 65 from Emcare and 151 from Medline. After removal of duplicates, 84 studies were screened, out of which 62 full-text articles were assessed for eligibility. 40 were excluded as they did not report on mortality specifically, leaving a total 22 studies to be included, as shown by the PRISMA diagram in Fig. 1. Studies reporting COVID-19 positive patients only, as well as those comparing them to a COVID-19 non-positive cohort and a pre-pandemic cohort were included. Grey literature such as conference abstracts, non-peer reviewed articles or letters were not included due to concern over the quality of rapid research work produced during the pandemic. There is a need for robust peer-reviews and strict measures to ensure that integrity of evidence synthesis is maintained^27^.

### Study characteristics and methodological assessment (Newcastle–Ottawa Scale)

No randomized-controlled trials were found during the search period. All studies included in this review were case–control studies and observational cohort studies, with seven of prospective^28–34^ and fifteen of retrospective^7,13,35–46^ design. The Newcastle–Ottawa scale (NOS) was used to assess methodological study quality, with a range of 1–9 (poor to good), as shown in Supplementary Table. All included studies in this review were susceptible to selection and timing bias due to the non-standardised COVID-19 testing protocols and inconsistent study periods whereby included patients may not be truly representative of true prevalence. The study characteristics are summarised in Table 1.

**Table 1:** Shows the characteristics of included studies.

Study	Study type	Study design	Country	Study period	Sample size	COVID-19 positive patients (n)	Population: age	Fracture type	Management	Outcomes	Reported mortality	Time of outcome assessment	NOS quality index
**Studies reporting COVID-19 positive patients only**													
Catellani 2020^28^	Single centre	Prospective observational	Italy	NR	16	16	85 (74–90)	Extra-capsular (11) Intracapsular (5)	IMHS (8) HHA (5)	Mortality POC	Pre-op 3 (18.8%) Post-op 4 (25%)	7 days in-patient	6
Cheung 2020^13^	Singles centre	Retrospective	USA	1 Mar–22 May	10	10	> / = 60	Extracapsular (10)	CMN (7) HHA (2) IF (1)	Mortality POC LOS	1 (10%)	Unclear	4
De 2020^35^	Multi-centre	Retrospective	UK	1 Mar–31 May	34	34	85.9 (SD 7.7)	Extracapsular (16) Intracapsular (18)	DHS (10) HHA (16) IMHS (6) THR (1) Conservative (1)	Mortality POC LOS	14 (41.2%)	30 days	7
Dupley 2020^36^	Multi-centre	Retrospective	UK	1 Mar–26 April	64	64	> / = 60 YO	Extracapsular (28) Intracapsular (36)	DHS (20) IMHS (8) CHS (1) HHA (29) Conservative (6)	Mortality POC LOS	21 (32.8%)	30 days	6
Jannelli 2020^29^	Single centre	Prospective	Italy	21 Feb–23 Mar	11	11	82.1 (59–95)	Extracapsular (6) Intracapsular (5)	Surgical (8) Conservative (3)	Mortality POC	2 (18.1%)	30 days	7
Morelli 2020^37^	Single centre	Retrospective	Italy	17 Mar–17 Apr	10	10	83.9 (72–98) (SD 7.4)	Extracapsular (10)	IMHS (8) HHA (2)	Mortality POC LOS	2 (20%)	?30 days (max 39)	5
Mi 2020^38^	Multi-centre	Retrospective	China	1 Jan–27 Feb	7	7	69.7 (34–85)	Extracapsular (5) Intracapsular (1) Femoral (1)	Surgical (3) Conservative (4)	Mortality	3 (42.9%)	In-patient	5
**Studies reporting COVID-19 positive patients compared to non-COVID-19 positive patients**													
Egol 2020^39^	Multi-centre	Retrospective	USA	1 Feb–Apr 15	138	17 (12.3%) *14 susp 107 neg	82.9 (SD 10.1)	Extracapsular (85) Intracapsular (71)	IMHS (5 vs 54) (5 suspected) HHA (5 vs 30) (7 suspected) THA (0 vs 6) SHS (0 vs 7) CRPP (3 vs 10) (2 suspected)	Mortality POC LOS	9 (52.9%) vs 6 (5.6%) 2 (14.3%) suspected	30 days	8
Fadulelmola 2020^40^	Multi-centre	Retrospective	UK	Mar–Apr	75	20 vs 55	83.5 (65–98)	Extracapsular (25) Intracapsular (50)	HHA (11 vs 36) DHS (6 vs 11) IMHS (2 vs 3) THR (0 vs 3) Conservative (1 vs 2)	Mortality POC	10 (50%) vs 4 (7.3%)	30 days	6
Hall 2020^41^	Multi-centre	Retrospective	UK	1 Mar–15 Apr	317	27 vs 290	> / = 50	NR	Fixation (15 vs 157) Arthroplasty (10 vs 121) Conservative (2 vs 12)	Mortality POC	9 (33.3%) vs 24 (8.3%)	30 days	8
Kayani 2020^7^	Multi-centre	Retrospective	UK	1 Feb–20 Apr	422	82 vs 340	> / = 18	Extracapsular (22 vs 68) Intrascapular (60 vs 272)	IMHS (14 vs 32) DHS (9 vs 36) THA (10 vs 37) HHA (42 vs 189) C-S (7 vs 46)	Mortality POC LOS	25 (43.9%) vs 35 (45.3%)	30 days	8
Konda 2020^30^	Multi-centre	Prospective	USA	Feb 1–April 15	319 (4th Q) (TOTAL	31 vs 288	> / = 55	NR	IMHS (10 vs 678) SHS (0 vs 89) HHA (12 vs 286) THA (0 vs 79) CRPP (5 vs 97) Conservative (4 vs 18)	Mortality POC LOS	11 (35.5%) vs 24 (8.3%)	30 days	8
LeBrun2020^42^	Multi-centre	Retrospective	US	20 Mar–24 Apr	59	9 vs 40 (10 NT)	> / = 65	Extracapsular (6 vs 30) Intracapsular (3 vs 20)	CRPP (1 vs 4) HHA (2 vs 11) THA (0 vs 2) CMN (4 vs 32) ORIF (0 vs 1) Conservative (2 vs 0)	Mortality POC	6 (66.7%) vs 1 (2.5%)	14 days	7
Malik 2020^43^	Single centre	Retrospective	UK	23 Mar–11 May	68	1 vs 67	84.3	Extracapsular (24) Intracapsular (44)	C-S (3) DHS (12) IMHS (11) HHA (39) THA (1) Conservative (2)	Mortality POC LOS	1 (100%) vs 5 (7.5%)	30 days	7
Maniscalco 2020^44^	Multi-centre	Retrospective	Spain	22 Feb–18 Apr	121	32 vs 32 (57 NT)	81.2	Extracapsular (69) Intracapsular (51) Periprosthetic (1)	THA (11) HHA (21) C-S (14) Blade-plate (1) IMHS (73)	Mortality	14 (43.8%) vs 1 (3.1%) (2 (3.5%))	21 days	6
Narang 2020^31^	Multi-centre	Prospective	UK	1 Mar–30 April	682	86 vs 596	86 vs 83	Extracapsular (46 vs 232) Intracapsular (38 vs 351)	NR	Mortality	30 (34.9%) vs 36 (6%)	30 days	9
Nunez 2020^45^	Single centre	Retrospective observational	Spain	Mar 14–April 02	512	99 vs 413	All hips: 88.4(SD 9.2)	NR	NR	Mortality	4 (4%)	20 days	5
Segarra 2020^32^	Single center	Prospective	Spain	Feb 1–Apr 15	68	2 vs 66	> 65	NR	Surgical (64) Conservative (4)	Mortality LOS	1 (50%) vs 4 (6.1%)	Mean 69.7 days	9
Slullitel 2020^46^	Single centre	Retrospective	Argentina	Dec 19–May 20	160	0 vs 74	86 (79–91)	Extracapsular (82) Intracapsular (78)	C-S (13) HHA (34) THA (29) IMHS (82) Girdlestone (1) Conservative (1)	Mortality LOS POC	0 vs 8 (10.8%)	30 days	5
Sobti 2020^33^	Single Centre	Prospective	UK	1 Mar–31 May	94	6 vs 47 (41 NT)	83.52	NR	NR	HHA (47) Fixation (35)	3 (50%) vs 5 (10.6%) (1 NT (2.4%))	NR	6
Thakrar 2020^34^	Single centre	Prospective	UK	15 Mar–15 Apr	43	12 vs 6 (25 NT)	81.6 (54–100)	NR	DHS (7) C-S (3) HHA (15) IMHS (13) THA (1) RHR (4)	Mortality	4 (33.3%) vs 1 (16.7%) (2 NT (8%))	30 days	8
Vives 2020^47^	Multi-centre	Retrospective	Spain	14 Mar–4 April	136	23 vs 39 (74 NT)	> / = 65	Extracapsular (84) Intracapsular (52)	Surgical (124) Conservative (12)	Mortality POC	7 (30.4%) vs 4 (10.3%) (2 NT (27%))	30 days	7

*POC* Post-operative complications, *LOS* length of stay, *DHS* dynamic hip screw, *C-S* cannulated hip screw, *HHA* hip hemiarthroplasty, *IMHS* intra-medullary hip screw, *THA* total hip arthroplasty, *RHR* revision hip replacement, *CRPP* closed-reduction percutaneous pinning, *IF* internal fixation, *CMN* cephalomedullary nail, *NR* not reported.

### Risk of bias

**Figure 2:**
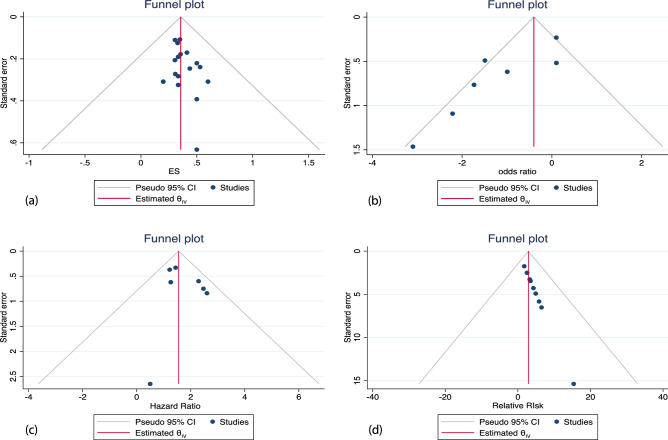
(**a**) Shows the funnel plot for case fatality rate. (**b**) Shows the funnel plot for excess mortality. (**c**) Shows the funnel plot for 30-day mortality. (**d**) Shows the funnel plot for excess mortality.

The distributions of calculated effect sizes were plotted against the precision of each study (standard error) on funnel plots which were found to be symmetrical, as shown in Fig. 2a–d. Furthermore, there was no evidence of publication bias due to small study effects based on Egger’s test for all 3 meta-analysis models, i.e., case fatality rate, all-cause mortality and 30-day mortality, with p = 0.21, 0.17 and 0.36 respectively.

## Findings

### Patients’ characteristics in meta-analyses

**Figure 3:**
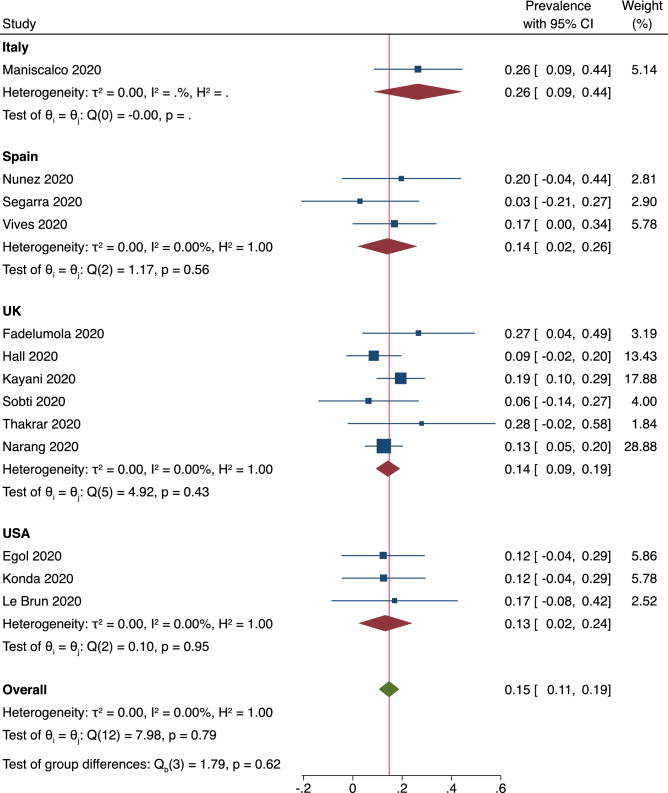
Shows the pooled prevalence of COVID-19.

The 22 papers included in the meta-analysis reported a total of 4015 patients. Out of these, 2651 (66.0%) were assessed during the COVID-19 period and 1364 (34.0%) were in the comparative pre-COVID-19 group. The mean age of included patients ranged from 57.5 to 86.1. The COVID-19 positive cohort of the meta-analysis population comprised of a total of 512 (19.3%) patients, of which 331 patients died during the study period. Pooled prevalence of COVID-19 was 15% [95% CI 0.11–0.19], as shown in Fig. 3. 2139 (80.7%) patients were not COVID-19 positive and 1017 (38.4%) were male hip fracture cases during the pandemic.

Based on pooled proportions, the most common medical comorbidities observed during the pandemic were dementia (37% [95% CI 31–43]), followed by diabetes (31% [95% CI 26–37%]), hypertension (20% [95% CI 14–26]), chronic lung disease (14% [95% CI 9–19%]), ischaemic heart disease (13% [95% CI 11–16%]) and chronic kidney disease (11% [95% CI 9–14%]). De et al.^35^ showed that COVID-19 positive patient who died had a higher CCI of 5.8 compared to those who were alive (5.3) whereas in Dupley et al.’s^36^ study, both groups had a similar CCI. Vives et al.^47^ observed a CCI ≥ 5 for deceased COVID-19 positive patients whilst four other studies^30,39,40,42^ have showed that patients with COVID-19 had a higher CCI than those who tested negative. When assessing frailty, four studies^7,31,40,41^ have shown that COVID-19 positive patients with a hip fracture had a higher NHFS, with a range of 5.2–6.0, as compared to those without COVID-19 (range 4.6–5.5).

Extracapsular fractures represented the greatest proportion of hip fractures (53% [95% CI 51–56]). Moreover, 99% [95% CI 98–99%] of these patients were managed surgically. The mean range for length of stay of patients during the COVID-19 period was 6.9–22.4 days^7,30,32,35,39,42,46^. Interestingly, seven studies^28,31,33,34,41,43,46^ reported the majority of patients from their cohorts having surgery within 36 h whilst the mean range of reported time to surgery was 24–72 h as shown in Table 2.

**Table 2:** Shows the patient characteristics for included studies.

Study	Mean age (years)	Gender M: F	Nursing home or facility living residence	Preoperative walking capacity and ADLs	ASA grade	Comorbidities (< 3 or > / = 3)/Charlson Comorbidity Index (CCI)	Known risk factors for COVID-19 mortality	Hip fracture prognostic scores (NHFS/CFS)	Time to surgery (h)	COVID-19 testing method	Type of anaesthesia	Length of stay	Discharge
**Studies reporting COVID-19 positive patients only**													
Catellani 2020^28^	85 (74–90)	10:6	NR	NR	NR	< 3 (9) > / = 3 (7)	DM (5) CKD (2) HTN (10) IHD (2)	NR	12–24 (10) 72 (3)*	CT RT-PCR (oropharyngeal swab)	Regional (16)	NR	NR
Cheung 2020^63^	79.7 (67–90)	2:8	NR	NR	NR	< 3 (1) > / = 3 (9)	HTN (7) COPD (1) DM (1) Dementia (1)	NR	46.7 (SD 39.7) vs 54.1 (SD 43.2)	RT-PCR (nasal and oropharyngeal swab)	Spinal (23) General (7) Spinal with block (2) General with spinal (1)	20.7 (SD 11.5) vs 22.4 (SD 11.8)	Home (1) Acute rehab (1) Subacute rehab (6)
De 2020^35^	84 (SD 7.7)	12:22	NR	NR	ASA-2 4 vs 1 ASA-3 11 vs 9 ASA-4 5 vs 4	CCI 5.3 (SD 1.4) vs 5.8 (SD 1.4)	NR	Frailty 5.85 (SD 1.5) vs 5.87 (SD 1.5)	NR	Serology	NR	NR	NR
Dupley 2020^36^	82 (SD 11)	29:35	NR	NR	ASA-1 1 ASA-2 5 ASA-3 32 ASA-4 19	CCI 6 vs 6	IHD (11) CCF (14) Dementia (27) COPD (12) DM (17)	NR	> 36 (4) < 24 (4)	RT-PCR	Regional or spinal (11)	NR	NR
Jannelli 2020^29^	86.1 (77–95)	1:10	NR	NR	ASA-2 2 ASA-3 6 ASA-4 2 N/A 1	< 3 (2) > / = 3 (9)	IHD (2) HTN (11) COPD (5) DM (3) Dementia (4)	NR	Days 2.7 (SD 3.9) [64.8 h] vs 1.1 (SD 0.6) [26.4]	RT-PCR Serology CT	General (7 vs 67) Spinal (6 vs 40)	9.8 (SD 5.2) vs 5.0 (SD 2.6)	NR
Morelli 2020^37^	83.9 (77–98)	2:8	NR	NR	NR	< 3 (5) > / = 3 (2) NR (3)	IHD (3) HTN (4)	NR	43.1 vs 38.3**	RT-PCR CT	General (11 vs 26) Spinal (8 vs 27)	NR	Rehabilitation unit (8)
Mi 2020^38^	69.7 (34–85)	2:5	NR	NR	NR	< 3 (6) > / = 3 (1)	HTN (3) DM (2) IHD (1) Dementia (1)	NR	< 36 h (17 vs 197) > 36 h (10 vs 80) N/A (6 vs 7)	RT-PCR	NR	NR	NR
**Studies reporting COVID-19 positive patients compared to non-COVID-19 positive patients**													
Egol 2020^39^	82.4 (SD 9.6) vs 83.4 (10.4)	16:15 vs 34:73	NR	NR	NR	CCI 2.1 (SD 1.8) vs 1.2 (SD 1.5)	IHD (54) COPD (16) DM (29) CKD (13) Dementia (34)	NR	72 h (2–5 days) vs 72 h (2–5 days)	RT-PCR	General (16 vs 58) Spinal (66 vs 282)	13.8 (SD 4.6) vs 6.7 (SD 2.5)	Home (9 vs 83)
Fadulelmola 2020^40^	83.7 vs 83.5	7:13 vs 15:40	NR	NR	NR	CCI 5.4 vs 5.1	NR	NHFS (6 vs 5.5)	NR	RT-PCR	NR	8.9d SD 6.8 vs 7.9 SD 4.8	Home (13 vs 38) Institution (7 vs 17)
Hall 2020^41^	83.6 (SD 11.3) vs 80.4 (10.6)	14:13 vs 92:198	Home (19 vs 211) Care/nursing home (6 vs 59) Hospital (2 vs 24)	NR	NR	NR	NR	NHS 5.3 (SD 1.7) vs 4.7 (SD 1.7)	29.9 h	RT-PCR	General (9) Spinal (46	10.8 days (7–29)	NR
Kayani 2020^7^	71.9 (SD 9.5) vs 72.7 (SD 6.7)	31:51 vs 136:204	Independent 2 vs 69 Package of care 15 vs 215 Residential home 39 vs 43 Nursing home 26 vs 13	Unaided 12 vs 38 One stick 41 vs 156 Two stick 25 vs 104 Frame 4 vs 35	ASA-1 3 vs 1 ASA-2 37 vs 172 ASA-3 36 vs 158 ASA-4 6 vs 9	< 3 57 vs 237 > / = 3 25 vs 103	NR	CFS 4.6 (SD 1.7) vs 5 (SD 1.9)	NR	RT-PCR (oropharyngeal swab) CT	NR	NR	Decline in social set-up 31 vs 62
Konda 2020^30^	NR	NR	NR	Ambulatory status COV+: 1.58 SD 0.7, COVID—1.33 SD 0.5	NR	CCI 1.9 SD 1.7 vs CCI 1.45 SD 1.7	IHD (9) CKD (35)	NR	< 36: 67 vs 344 > 36: 19 vs 237	RT-PCR (oro/nasopharyngeal swab)	NR	NR	3 vs 40
LeBrun2020^42^	86.5 (SD 7.9) vs 84.7 (SD 7.5)	3:6 vs 12:38	Home 5 vs 42 Nursing Home 2 vs 4 Assisted living 2 vs 3	Community ambulator without assist: 2 vs 23 Community ambulance with assist: 20 vs 10 Household ambulator with assist 24 vs 12 Bedbound/wheelchair: 8 vs 4 Unknown 2 vs 1	Mean ASA 3 vs 2	CCI 6.5 vs 5.7	IHD (34) CKD (21) Dementia (44) DM (43) CKD (15)	NR	NR	RT-PCR	NR	NR	Home: 0 vs 26 Skilled nursing facility: 3 vs 15 Hospice: 1 vs 1
Malik 2020^43^	84.3 (SD 8.9)	25:43	Nursing Home (8) Residential care (8) Own home (52)	NR	ASA 1 (0) ASA 2 (17) ASA 3 (47) ASA 4 (4)	NR	NR	NR	21.8 h	NR	NR	8.6 days	NR
Narang 2020^31^	86 vs 83	32:53 vs 169:424	NR	NR	3.3 vs 3.0	NR	NR	NHFS: 5.9 vs 5	1.8 day SD 1.3 vs 1.5 day SD 1.6	RT-PCR (oropharyngeal swab) Serology	NR	6.9 days SD 2.5 vs 6.3 days SD 2.4	NR
Nunez 2020^45^	57.5 (SD 22.5)	247:265	NR	NR	NR	NR	NR	NR	24d	RT-PCR	Spinal	6 days vs 5 days	Home (436 vs 1977) Hospital (66 vs 137) Flight (9 vs 42) Voluntary discharge (1 vs 5)
Segarra 2020^32^	82.4 (SD 7.4)	51:93	NR	NR	ASA 2 29 vs 22 ASA 3 37 vs 48 ASA IV 2 vs 0	NR	NR	NR	< 24 h (52 vs 74)	NR	NR	NR	NR
Slullitel 2020^46^	86	9:65	NR	NR	ASA ½–12 vs 22 ASA ¾–62 vs 64	CCI 1–2: 1 vs 3 CCI 3–4:13 vs 31 CCI 5: 59 vs 52	NR	Frailty: 42:32	< 36 h (26) > 36 h (17) Mean 51.2 (10.2–128.8)	RT-PCR (oro/nasopharyngeal)	NR	NR	NR
Sobti 2020^33^	83.52	NR	NR	NR	ASA ¾: 75 vs 80	NR	NR	NR	2.4 (± 2.2) (alive) vs 2.2 ± 2.3 (dead)	RT-PCR (oropharyngeal)	NR	NR	NR
Thakrar 2020^34^	81.6 (54–100)	23:20	NR	NR	NR	NR	NR	NHFS 5.2 (1–8) CFS 4.6 (1–7)	1.2 days	RT-PCR (nasopharyngeal)	General (3) Neuraxial (7)	7.8 days	NR
Vives 2020^47^	85 (65–101)	34:102	Home (106) Nursing home (30)	NR	ASA-1 2 ASA-II 13 ASA-III 88 ASA-IV 12 ASA-V 2	CCI > / = 5 (7) [COVID-19 positive deaths only]	NR	NR	NR	RT-PCR (nasopharyngeal) CT	NR	NR	NR
Maniscalco 2020^44^	81.2	9:8	NR	NR	NR	NR	NR	NR	NR	RT-PCR (nasopharyngeal swab) Chest-CT	NR	NR	NR

*NR* Not reported, *M* Male, *F* Female, *ADL* Activities of daily living, *ASA* American society of anaesthesiologists, *RT-PCR* Reverse transcriptase polymerase chain reaction, *CT* Computed tomography, *d* days, *SD* standard deviation.

### Deceased patients’ characteristics in meta-analyses

The pandemic witnessed a total of 331 deceased patients amongst the included studies. Out of these, 180 were infected with COVID-19, and 151 were tested as COVID-19 negative. The remaining 9 were either not tested or considered as suspected. The mean age range was 84–93.5. Amongst all deaths, 12 studies^7,13,28,29,32,35–38,41,42,47^ differentiated based on patient gender with 55.4% of deceased patients being females, reflecting the higher number of female elderly patients. During the pandemic, the greatest proportion of deceased patients presented with extracapsular fractures (85% [95% CI 69–96%]). Moreover, 76% [95% CI 66–86%] of all deceased underwent operative management for their injury, where 44% (95% CI 31–58%) had surgical fixation.

Amongst deceased hip fracture patients with COVID-19 infection, the greatest number of fractures were extracapsular (68% [95% CI 53–81%]) and an intra-medullary device was the most commonly used implant 54% [95% CI 35–73%]. Additionally, hypertension (60% [95% CI 0.25–0.91]), diabetes (57% [95% CI 0.37–0.76]) and dementia (29% [95% CI 0.11–0.49]) were the most frequently observed comorbidities in COVID-19 positive hip fracture deaths.

The baseline characteristics and demographics of deceased patients are shown in Table 3.

**Table 3:** Characteristics of deceased patients.

Study	N	COV+	COV−	Age (Cov+ vs –) (years)	M:F (Cov+ vs cov−)	Co-morbidity (Cov+ vs –)	Place of residence (Cov+ vs −)	ASA Grade (Cov+ vs –)	Prognostic score (CFS/NHFS) (Cov+ Cov−)	Fracture type	Management	Time to surgery	Admission to death	Surgery to death	LOS
**Studies reporting number of deaths for COVID-19 positive patients only**															
Catellani 2020^28^	7	3 Pre-op 4 Post-op	0	83.1	4:3	< 3–3 > / = 3–2	NR	NR	NR	5 EC 2 IC	2 IMHS 2 HHA	NR	3.5 days	NR	NR
De 2020^35^	14	14	0	88.8	8:6	CCI 5.8 SD 1.4	NR	ASA 2-1 ASA 3-9 ASA 4-4	CFS 5.87 (SD 1.5) NHFS 6.2 (SD 0.9)	10 EC 4 IC	4 DHS 4 HHA 5 IMHS 1 Conservative	54.1 h (SD 43.2)	NR	NR	22.4 (SD 11.8)
Jannelli 2020^29^	2	2	0	89 (range 86–92)	0:2	> 3 = 2	NR	ASA 3-2	NR	1 IC 1 EC	2 Operative	NR	NR	NR	NR
Morelli 2020^37^	2	2	0	93.5 (range 89–98)	0:2	< 3 = 2	NR	NR	NR	2 EC (31-A)	2 Operative	NR	16.5 days (range 15–18)	8 days	9 days
Cheung 2020^13^	1	1	0	NR	0:1	> 3–1	NR	NR (1 GA)	NR	1 EC (31-A)	1 CMN	NR	NR	19 Days	NR
Mi 2020^38^	3	3	0	81.7 (76–85)	2:1	< 3 = 3	NR	NR	NR	3 EC	1 Operative 2 Conservative	NR	20.3 days (8–39)	11 days	NR
Dupley 2020^36^	21	21	0	84 (SD 6)	12:9	CCI 6 (SD 2)	NR	NR	NR	NR	17 Operative 4 Conservative	NR	NR	NR	NR
**Studies reporting total number of deaths for COVID-19 positive and non-COVID-19 positive patients**															
Egol 2020^39^	15	9	6 (2 suspected)	NR	NR	NR	NR	NR	NR	NR	NR	NR	NR	NR	NR
Fadulelmola 2020^40^	14	10	4	NR	NR	NR	NR	NR	NR	NR	NR	NR	NR	NR	NR
Hall 2020 (Total deaths)^41^	33	9	24	85.8 (SD 7.9)	18:15	NR	Home—18 Care home—11 Hospital—4	ASA 2-3 ASA 3-16 ASA 4-8 ASA 5-1 NR-5	5.8 (SD 1.4)	NR	16 Fixation 10 Arthroplasty 7 Conservative	< 36–17 > 36–10 NR-6	NR	NR	NR
Kayani 2020 (Total deaths (Cov + deaths))^7^	60	25	35	NR	31:51 (9:16)	< 3 57 (4) > 3 25 (21)	Independent 17 (7) Residential 65 (18)	ASA 1,2 40 (12) ASA3,4-42 (13) GA 16 (5) SA 66 (20)	NR	IC 60 (17) EC 22 (8)	42 (11) HHA 10 (4) THR 9 (4) DHS 14 (4) IMHS 7 (2) C-S	NR	NR	NR	NR
Konda 2020^30^	35	11	24	NR	NR	NR	NR	NR	NR	NR	NR	NR	NR	NR	NR
LeBrun 2020 (Total deaths (COV+ vs –))^42^	7	6	1	86.7 (85.8 vs 92)	3:4 (3:3 vs 0:1)	> 3–7 (6 vs 1)	NR	NR	NR	7 EC (6 vs 1)	4 (4 vs 0) CMN 1 (0 vs 1) HHA 2 (2 vs 0) Conservative	NR	NR	7.6 (7.6 vs 0)	NR
Malik 2020^43^	6	1	5	NR	NR	NR	NR	NR	NR	NR	NR	NR	NR	NR	NR
Maniscalco 2020^44^	17	14	1 (2NT)	NR	NR	NR	NR	NR	NR	NR	NR	NR	NR	NR	NR
Narang 2020^31^	66	30	36	NR	NR	NR	NR	NR	NR	NR	NR	NR	NR	NR	NR
Nunez 2020^45^	4	NR	NR	NR	NR	NR	NR	NR	NR	NR	NR	NR	NR	NR	NR
Segarra 2020 (COV+ only deaths)^32^	5	1	4	88	0:1	NR	Nursing home—1	ASA 3-1	NR	NR	1 Operative	NR	NR	NR	NR
Slullitel 2020^46^	8	0	8	NR	NR	NR	NR	NR	NR	NR	NR	NR	NR	NR	NR
Sobti 2020^33^	9	3	5 (1 NT)	NR	NR	NR	NR	NR	NR	NR	NR	NR	NR	NR	NR
Thakrar 2020^34^	7	4	1 (2 Not tested)	NR	NR	NR	NR	NR	NR	NR	NR	NR	NR	NR	NR
Vives 2020 (Total deaths (COV + ve only))^47^	13	7	4 (2 NT)	87 SD 7.2 (91.2)	5:8 (3:4)	(CCI 6.6)	Nursing home 7 (5) Own home—6 (2)	ASA 3-7 (4) ASA 4-2 1) ASA 5-5 (1) NR (1)	NR	IC 4 (1) EC 9 (6)	5 Operative (2 × IMHS) 8 conservative	2.2 days SD 3 (4 days)	7.5 days SD-5 (NR)	NR	7.5 days SD 5 (NR)

*N* Number of patients, *COV+* COVID-19 Positive patients, *COV–* non-COVID-19 patients, *ASA* American society of anaesthesiologists, *LOS* length of stay, *NR* Not reported, *SD* Standard deviation, *CFS* Clinical frailty score, *NHFS* Nottingham hip fracture score, *IMHS* Intramedullary Hip screw, *HHA* Hip hemiarthroplasty, *DHS* Dynamic hip screw, *THA* Total hip arthroplasty, *CMN* Cephalomedullary nail, *EC* Extracapsular, *IC* Intracapsular.

### Mortality meta-analyses

#### Excess mortality

##### All hip fracture patients during COVID-19 period vs pre-COVID-19 period

**Figure 4:**
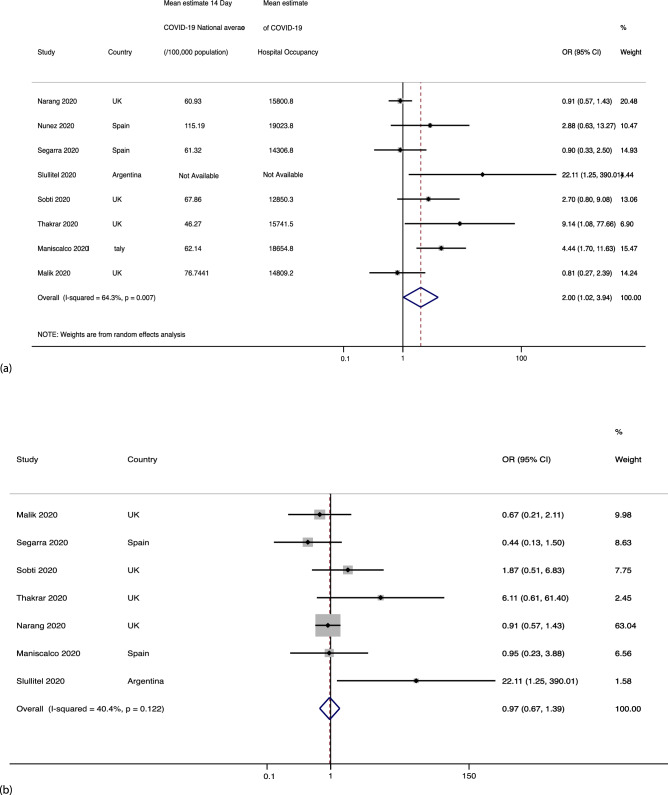
(**a**) Shows the forest plot for all hip fracture patients during the COVID-19 period vs pre-COVID-19 period. (**b**) Shows the forest plot for non-positive COVID-19 patients during the pandemic vs pre-COVID-19 control.

Eight studies^31–34,43–46^ reported mortality during the pandemic compared to a pre-COVID-19 control group. A higher mortality was observed during the pandemic of all hip fracture patients (10% [95% CI 0.08–0.11]) in comparison to the control group 5% [95% CI 0.04–0.06]), p < 0.001. From the included studies pooled estimates obtained indicated an increased hip fracture mortality during the COVID-19 pandemic, OR 2.00 [95% CI 1.02–3.94], p = 0.007, I^2^ = 64.3%, as shown in Fig. 4a. All the studies reporting excess mortality were considered as moderate or good quality studies, and hence sensitivity analyses remained unchanged^31–34,43–46^.

##### Non-positive COVID-19 patients during the pandemic vs pre-COVID-19 control

Seven studies^31–34,43,44,46^ reported the number of non-COVID positive deaths during the pandemic period, which was meta-analysed against the mortality in the pre-COVID-19 control cohort. An odds ratio (OR) of 0.97 (95% CI 0.67–1.39), p = 0.08 was observed, as shown in Fig. 4b.

#### All-cause mortality (All reported deaths of COVID-19 positive vs non-positive COVID-19 patients)

**Figure 5:**
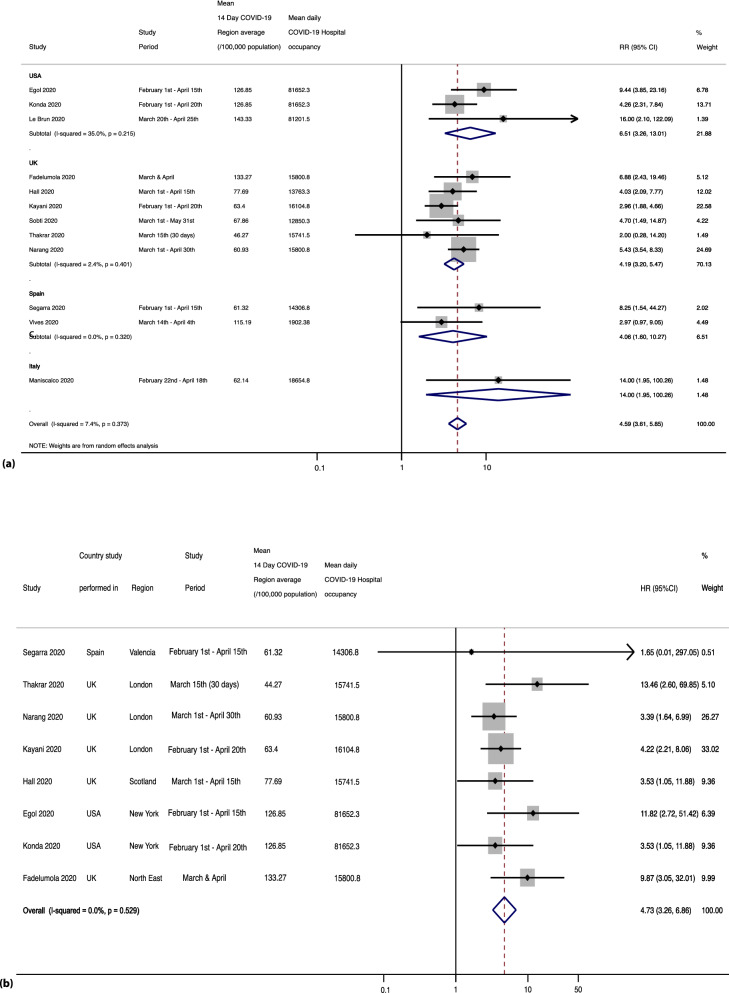
(**a**) Shows the forest plot for all-cause mortality (all reported deaths of COVID-19 positive vs non-positive COVID-19 patients). (**b**) Shows the forest plot for 30-day mortality for COVID-19 positive vs non-positive COVID-19 patients.

Twelve studies^7,30–34,39–42,44,47^ reported mortality in COVID-19 positive hip fracture patients against a non-positive COVID-19 cohort. Within this cohort, COVID-19 positive deaths were 47% (95% CI 0.40–0.53) whilst COVID-19 non-positive deaths were 53% (95% CI 0.46–0.59). A relative risk (RR) of 4.59 [95% CI 3.61–5.85], p < 0.0001, [I^2^ 7.4%, p = 0.373], Z = 12.39 showed increased risk of hip fracture mortality due to COVID-19 infection, as shown in Fig. 5a. Greatest risk ratio was seen in studies from the USA, RR 6.51 (95% CI 3.26–13.01), p < 0.0001). Pooled estimates for UK-based studies was RR 4.19 (95% CI 3.20–5.47), p < 0.0001. All-cause mortality within the Spanish studies was similarly found to be RR = 4.06 (95% CI 1.60–10.37), p = 0.003, and a single study from Italy was RR = 14 (95% CI 1.95–100.26), p = 0.009. Sensitivity analysis assessing all-cause mortality remained unchanged as all studies were of moderate or good quality^28–37,39,39–45,47^.

#### 30-day mortality for COVID-19 positive vs non-positive COVID-19 patients

Eight studies^7,30–32,34,39–41^ assessed the impact of COVID-19 positive status on 30-day mortality of hip fracture patients showing a 38% (95% CI 0.32–0.44) 30-day mortality for COVID-19 positive patients in comparison to 7% (95% CI 0.06–0.08) for non-COVID-19 positive patients. We performed a time-to-event meta-analysis using an inverse variance random effects model to obtain a pooled hazard ratio HR of 4.73 (95% CI 3.26–6.86) [Z = 8.19, p < 0.0001] as shown in Fig. 5b. Variation in the pooled HR attributable to heterogeneity was low, I^2^ = 0.0%, p = 0.529. Studies that reported 30-day mortality were of moderate or good quality, and thus sensitivity analysis was unchanged^7,30–32,34,39–41^.

#### Case fatality rate (CFR) for COVID-19 positive patients

**Figure 6:**
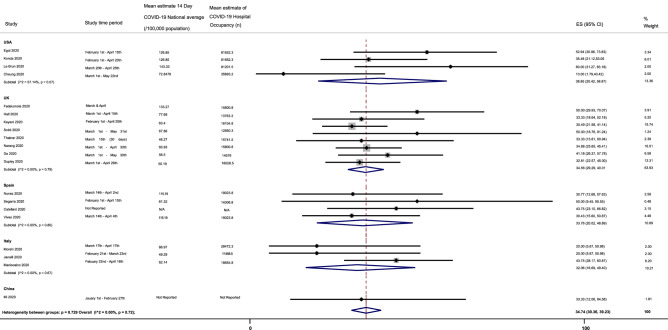
Shows the forest plot for case fatality rates (CFR) for COVID-19 positive patients.

The pooled estimate for case fatality rate (CFR) observed for hip fracture patients and concomitant COVID-19 infection was 34.74% (95% CI 30.36–39.23) [I^2^ = 0.00, p = 0.72], as shown in Fig. 6. The CFR of individual countries was further analysed during the pandemic to reveal values of 38.85% (95% CI 20.42–58.87) [z-value 5.71] for the USA, 34.56% (95% CI 29.29–40.01) [z-value 5.23] for the UK, 33.76% (95% CI 20.02–48.69) [z-value 6.69] for Spain, 32.06% (95% CI 16.69–49.40) [z-value 5.70] for Italy and 33.33% (95% CI 12.06–64.58) [z-value 2.91] for China. All values obtained were found to be significant, with a p < 0.001. Sensitivity analysis of only moderate to good quality studies^7,28–42,44,45,47^ revealed a similar in-patient mortality rate of 35.32% (95% CI 30.88–39.87) [I^2^ = 0.00, p = 0.85], with no statistical difference.

### Meta-regression

#### Impact of COVID-19 infection and hospital occupancy

**Figure 7:**
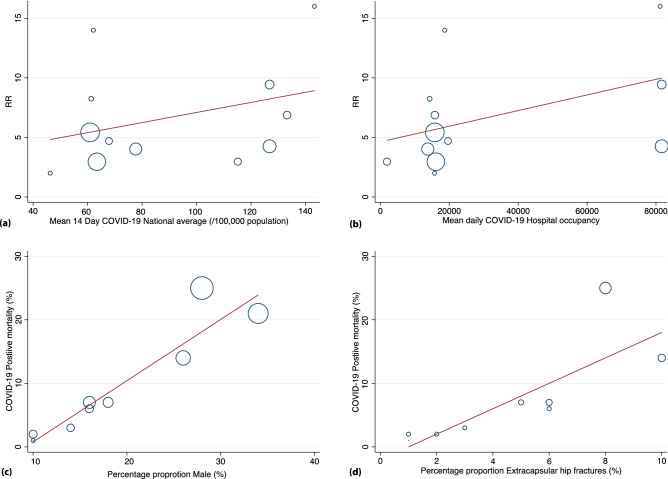
(**a**) Shows the meta-regression plot on the impact of 14-day COVID-19 prevalence. (**b**) Shows the meta-regression plot on the impact of mean daily COVID-19 hospital occupancy on pooled hip fracture mortality. (**c**) Shows the meta-regression plot on the impact of male proportions on COVID-19 positive hip fracture mortality. (**d**) Shows the meta-regression plot on the impact of proportions of extracapsular hip fractures on COVID-19 positive hip fracture mortality.

Meta-regression was performed to evaluate the impact of COVID-19 prevalence on pooled mortality estimates as shown in Table 4. Significance was seen between all-cause mortality, COVID-19 prevalence (p = 0.01) and hospital occupancy (p = 0.05) suggesting a positive association, as shown in Fig. 7a,b. No correlation or association was seen between 30-day mortality, prevalence and hospital occupancy due to COVID-19 infection.

**Table 4:** Shows the meta-regression table for the included studies.

	Covariates	Regression coefficient	SE	95% CI for coefficient	P value
Case fatality rate	14-day average COVID-19 positive cases during the time period	1.0012	0.0016	0.9977–1.0045	0.47
	Hospital occupancy with COVID-19 patients during the time period	1.0000	0.0967	0.9999–1.0000	0.47
All-cause mortality	14-day average COVID-19 positive cases during the time period	1.0510	0.2910	0.9859–1.1204	0.01
	Hospital occupancy with COVID-19 patients during the time period	1.0000	0.0003	0.9999–1.0001	0.05
30-day mortality	14-day average COVID-19 positive cases during the time period	1.0070	0.0064	0.9914–1.0228	0.31
	Hospital occupancy with COVID-19 patients during the time period	1.0000	0.0064	0.9998–1.0002	0.91

#### Predictors of mortality in hip fracture patients with COVID-19 infection

##### Gender

Of the nine studies^7,13,28,32,35,36,38,42,47^ reporting gender demographics amongst the COVID-19 patients, a positive association was seen between an increasing proportion of COVID-19 positive male hip fracture patients and mortality as shown in Fig. 7c, exp(b) = 6.87 (95% CI 3.73–12.64), p < 0.001.

##### Comorbidities

A positive association was found between an increasing proportion of COVID-19 patients with diabetes and dementia and hip fracture mortality, where exp(b) = 7.38 (95% CI 3.31–16.49), p = 0.002 and exp(b) = 40 (95% CI 25.57–65.21), p = 0.001, respectively.

##### Type of fracture and intervention

**Figure 8:**
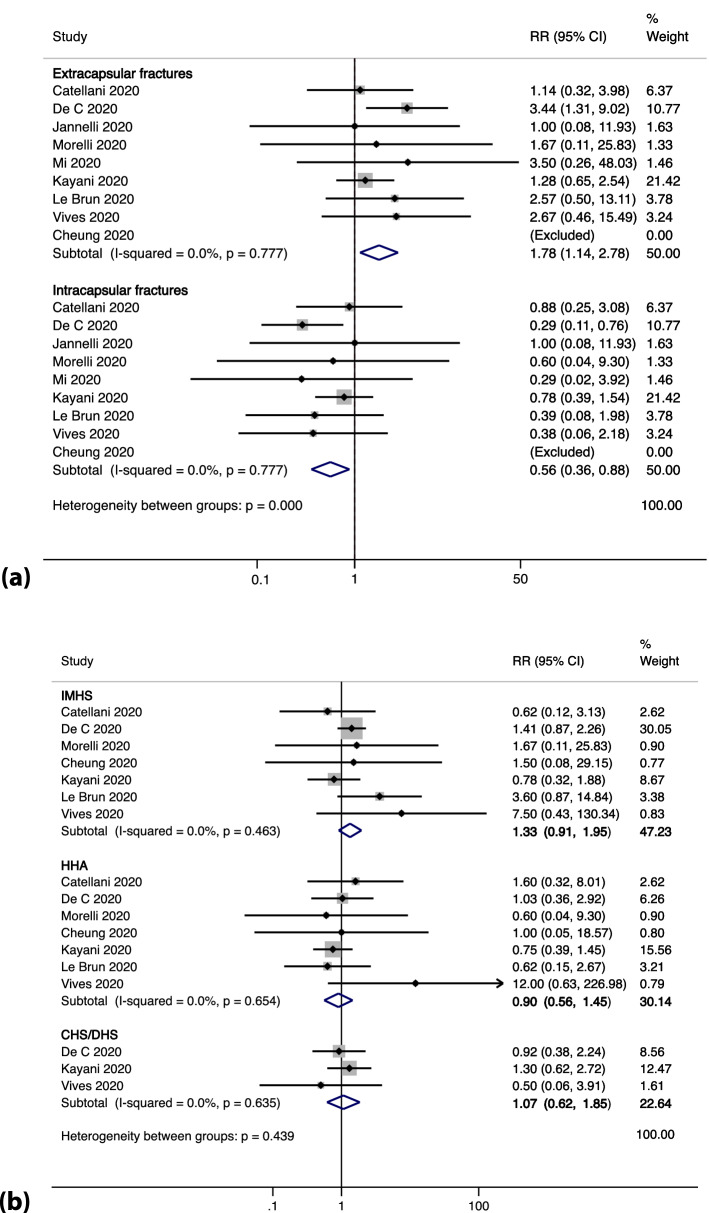
(**a**) Shows the forest plot for the type of hip fracture in COVID-19 positive deaths. (**b**) Shows the forest plot for type of implant in COVID-19 positive deaths.

Risk of mortality was greatest in COVID-19 positive patients with extracapsular fractures, RR 1.78 (95% CI 1.14–2.78), p = 0.012, as shown in Fig. 8a. Through meta-regression we observed an increasing proportion of such injuries to be predictive of worsening mortality amongst COVID-19 positive patients, exp(b) 7.40 (95% CI 1.56–35.03), p = 0.019, as shown in Fig. 7d. 27% of patients underwent fixation using an intramedullary device and pooled estimates RR 1.33 (95% CI 0.91–1.95), p < 0.04 suggest increased risk of mortality amongst the COVID-19 positive patients when using this implant, as shown in Fig. 8b.

## Discussion

This meta-analysis provides an in-depth review of the impact COVID-19 has had on the mortality of patients with hip fractures. We identified independent predictors of poor outcomes in hip fracture patients testing positive for COVID-19 and have demonstrated a four-fold increased risk of mortality in this cohort following admission (RR 4.59) and a 30-day mortality of 38% (HR 4.73). Moreover, the overall case fatality rate for COVID-19 positive hip fracture patients was 34.74% which is substantially higher than reported case fatality rates for patients with COVID-19 infection, ranging from 3.5 to 20.8% with increasing age^48^.

Despite various containment protocols and mitigation strategies adopted by countries during the pandemic, the incidence of hip fractures has remained unchanged^7,31^. Independent of COVID-19 infection, patients with hip fractures have a reported 30-day mortality of 7.5–10%^49^. Hospitalization of these patients may subject them to additional risk given their vulnerability to COVID-19 in an overburdened healthcare system, potentially resulting in suboptimal healthcare provision^39,40,42^. This is apparent as we observed a mortality of 10% in all hip fractures, with a two-fold increase (OR 2.00, p = 0.007) in excess mortality. Whilst this rise may theoretically be due to the indirect impact of the pandemic irrespective of the patients’ COVID-19 positive status, our analysis has shown no overall increased risk when comparing non-positive COVID-19 patients to the pre-pandemic cohort (OR 0.97, p = 0.08). This suggests that having a COVID-19 infection may independently impact the excess mortality observed.

With uncertainty around the novel variants and lack of definitive treatment, the need to enforce measures to reduce the spread of this disease is essential to mitigate mortality. Through our meta-regression, we observed that prevalence of COVID-19 disease (number of positive cases/100,000) and hospital occupancy due to COVID-19 directly affect the all-cause mortality of patients with hip fractures (p = 0.01 and 0.05 respectively). This highlights the concern that a non-linear rise in mortality risk may be seen if tight infection control measures are not implemented, due to healthcare systems being overwhelmed by critically unwell patients. We observed a high pooled case fatality rate (38.9%) from studies performed in the USA, specifically New York, with a sixfold increased risk of mortality in COVID-19 positive hip fractures. This may be explained by the region being the epicentre of the pandemic and reflected by the high prevalence of COVID-19 infection and associated hospital occupancy further supporting the impact this pandemic has had through straining of healthcare resources^50^.

The pre-morbid status of patients has been shown to independently contribute to adverse outcomes in patients with an isolated hip fracture or COVID-19 infection. The vulnerability of these groups subjects them to a far greater risk of poor outcomes, as highlighted in the included studies^7,35,36,39,41,47^. Known predictors such as advanced age, male gender, frailty, multiple comorbidities, dementia and cognitive impairment, ASA grade (American Society of Anesthesiologists), baseline ambulation and residential status are well established risk factors of mortality in hip fracture patients^51^. Many of these risk factors overlap with known predictors of COVID-19 mortality from recent studies^52^. Our findings have reflected this through a positive association seen between hip fracture mortality, male gender (p < 0.001), diabetes (p = 0.002) and dementia (p = 0.001), which to our knowledge are novel findings.

Recent studies have suggested that the mortality of COVID-19 patients may be due to the virally driven cytokine storm response^53,54^ which subjects patients to an increased risk of thromboembolic events and could exacerbate the hypoxaemia seen in COVID-19 related acute respiratory distress syndrome (ARDS)^55,56^. A similar cytokine mediated inflammatory response has been studied in patients with hip fractures, where the cytokine kinetics curves were higher in patients with worsened outcomes^57^. This supports the “two-hit theory” proposed by various studies^40,42,58^ whereby the pro-inflammatory state induced by the stress of injury, coupled with a “second-hit” resulting from surgical insult may exacerbate inflammation in acutely ill COVID-19 patients. Whilst this might skew the decision towards conservative management, our study showed that patients who had surgical repair still had a more favourable outcome. Another factor weighing into this decision is the time to surgery. Whilst the recommended timeframe for surgery is within 36 h, six studies^7,32,35,39–41,47^ reported a time to surgery > 36 h.

Seven studies^7,28,31,35,36,42,47^ reported a higher mortality with extracapsular fractures amongst patients with COVID-19 which is supported by our pooled estimate (RR 1.78, p = 0.012). This is in line with established evidence of the poorer outcomes observed for such injuries as patients susceptible to extracapsular fractures are often older, with hip osteoarthritis requiring osteosynthesis^59^. This leads to a slower recovery, longer length of hospital stay with an increased risk of nosocomial infections, and prolonged surgical procedures in unstable injuries^51^. Additionally, four studies^7,35,42,47^ reported an increased mortality with intramedullary fixation, as shown in our results (RR 1.33, p = 0.04) in patients with COVID-19 infection. The obvious difference between this type of implant and other forms of extracapsular fixation is the instrumentation of the femoral canal which is known to be associated with increased mortality due to increased intramedullary pressure, embolic showers and fat extravasation and may be catastrophic to COVID-19 patients, representing a “second-hit” postulated by Lebrun et al. and Egol et al.^39,42,60,61^.

The change in theatre organisation, with the appropriation of additional steps to accommodate aerosol generating procedure (AGPs), has resulted in an increase in operative delay over the COVID-19 period, as shown by Narang et al. and Segarra et al.^31,32^. The former^31^ has however observed a faster time to surgery for COVID-19 infected patients, possibly due to a conscious decision to expedite surgery in an attempt to improve outcomes for these patients. The benefits of early intervention (within 36 h) is well-known in the literature^62^. To overcome the hurdles imposed by COVID-19, Malik et al.^43^ implemented an multidisciplinary (MDT) approach to facilitate decision making, resulting in a reduced COVID-19 mean time to surgery compared to pre-COVID-19 era (21.8 h vs 28.2 h) as well as a shortened time from admission to orthogeriatric assessment.

This apparent discrepancy between prevalence and mortality in our analysis might be due to missed opportunities to identify and prioritise management of COVID-19 positive hip fracture patients who are in the highest risk cohort. Infectivity is dynamic and being in hospital increased the risk of viral transmission. Whilst the aim should be to avoid prolonged inpatient stay, the health-burden of COVID-19 may not necessarily allow this. Supporting this, five authors^7,32,39,42,46^ observed an increased mean LOS. However, by carefully risk stratifying patients and deciding the management plan, Malik et al. showed a statistically reduced LOS of patients during the pandemic, thus minimising risks involved with transmission (8.6 vs 16.3).

There are several limitations to our meta-analysis. The studies included ranged from case series to multicentre studies with varying testing protocols which may affect the true representativeness of the study population limiting the conclusions drawn in this review. Only studies in English were included which may introduce further selection bias. Moreover, the majority of studies were performed in Europe, and this imbalance of sources increases the possibility of publication bias. In several studies, clinical parameters were not clearly defined in addition to varying follow-up times. Moreover, we observed heterogeneity in the range of symptoms, interventions and outcomes reported amongst the studies included due to a lack of objective measurements. Despite these limitations, our study is the first to quantify the independent impact of COVID-19 infection on hip fracture mortality. Furthermore, we have identified modifiable variables through our analysis which can impact outcomes for vulnerable patients potentially enabling a better surgical risk stratification. Hence, there is a requirement for more robust evidence through larger samples and more reliable testing methods to further establish the true impact of COVID-19 on hip fracture outcomes.

## Conclusion

Our study has shown an increased overall and 30-day mortality of hip fracture patients treated during the COVID-19 pandemic with concomitant COVID-19 infection being an independent risk factor of mortality. We highlight the impact prevalence and hospital occupancy has had on mortality as surrogate markers of overburdened healthcare systems. We believe the vulnerability of hip fracture patients increases with peak incidence of COVID-19. Thus, their care must be prioritised during this crisis through means of a comprehensive care pathway. Furthermore, we identified modifiable predictors of poor outcomes in COVID-19 positive hip fractures, such as male gender, diabetes, dementia and intramedullary fixation devices, enabling clinicians to mitigate risk and aid decision-making during this pandemic.

## Supplementary Information

Supplementary Table.

## Data Availability

Data and data sets for this study are available from the corresponding author upon reasonable request.
