# Supplementary material for: Meta-analysis and metaregression of risk factors associated with mortality in hip fracture patients during the COVID-19 pandemic
Source: Sci Rep. 2021 May 12;11:10157. doi: 10.1038/s41598-021-89617-2 (PMC8115062; doi:10.1038/s41598-021-89617-2)
Supplement: Supplementary file 1 — Supplementary Table. [file 41598_2021_89617_MOESM1_ESM.docx]

**META-ANALYSIS AND METAREGRESSION OF RISK FACTORS ASSOCIATED WITH MORTALITY IN HIP FRACTURE PATIENTS DURING THE COVID-19 PANDEMIC**

Firas J. Raheman*^1^, MBBS, MRCS, MAcadMed, PGDip

Djamila M. Rojoa^1^, MB ChB

Jvalant Parekh^1^, MB ChB

Reshid Berber^2^, MBBS, BSC(Hons), FRCS(Tr&Orth), PhD

Robert Ashford^1,3^, MBBS, MD, FRCS(Tr&Orth)

^1^ The Leicester Royal Infirmary, University Hospitals of Leicester, UK

^2^ Nottingham University Hospitals, NHS Trust, UK

^3^ Leicester Cancer Research Centre, University of Leicester, UK

| Supplementary table. Shows the methodological quality assessment using the Newcastle-Ottawa Quality Assessment Scale (NOS) for case-control and cohort studies. Studies were awarded a maximum of one star for each numbered item within the Selection and Outcome categories. A maximum of two stars were given for Comparability. | | | | | | | | | |
| --- | --- | --- | --- | --- | --- | --- | --- | --- | --- |
| **Studies** | **Selection** | | | | **Comparability** | **Exposure** | | | **Total Quality Score** |
| **Author, year** | **Representativeness of the exposed cohort** | **Selection of non-exposed cohort** | **Ascertainment of exposure** | **Demonstration of outcome of interest was not present at start of study** | **Comparability of cohorts on the bases of the designs or analysis** | **Assessment of outcome** | **Was follow-up long enough for outcomes to occur** | **Adequacy of follow up of cohorts** |  |
| **Case-series reporting on COVID-19 positive patients only** | | | | | | | | | |
| Catellani 2020 (27) | * | None | * | * | ** | * | - | - | 6 |
| Cheung 2020 (62) | * | None | * | - | * | * | - | - | 4 |
| De 2020 (34) | * | None | * | - | ** | * | * | * | 7 |
| Dupley 2020 (35) | * | None | * | - | * | * | * | * | 6 |
| Jannelli 2020 (28) | * | None | * | * | * | * | * | * | 7 |
| Morelli 2020 (36) | * | None | * | * | * | * | - | - | 5 |
| Mi 2020 (37) | * | None | * | - | * | * | - | - | 5 |
| **Cohort studies reporting on COVID-19 positive patients compared to non-COVID-19 positive patients** | | | | | | | | | |
| Egol 2020 (38) | * | * | * | - | ** | * | * | * | 8 |
| Fadulelmola 2020 (39) | * | - | * | - | * | * | * | * | 6 |
| Hall 2020 (40) | * | * | * | - | ** | * | * | * | 8 |
| Kayani 2020 (7) | * | * | * | - | ** | * | * | * | 8 |
| Konda 2020 (29) | * | * | * | * | * | * | * | * | 8 |
| LeBrun2020 (41) | * | * | * | - | ** | * | - | - | 7 |
| Malik (42) | - | * | * | * | * | * | * | * | 7 |
| Maniscalco (43) | * | * | * | * | * | * | - | - | 6 |
| Narang 2020 (30) | * | * | * | * | ** | * | * | * | 9 |
| Nunez 2020 (44) | * | * | * | - | * | * | - | - | 5 |
| Segarra 2020 (31) | * | * | * | * | ** | * | * | * | 9 |
| Slullitel 2020 (45) | - | * | - | - | * | * | * | * | 5 |
| Sobti 2020 (32) | * | * | * | * | * | * | - | - | 6 |
| Thakrar 2020 (33) | * | * | * | * | * | * | * | * | 8 |
| Vives 2020 (46) | * | * | * | - | * | * | * | * | 7 |
